# Cytokinin and reproductive shoot architecture: bigger and better?

**DOI:** 10.1042/BST20231565

**Published:** 2024-07-31

**Authors:** Catriona H. Walker, Tom Bennett

**Affiliations:** School of Biology, Faculty of Biological Sciences, University of Leeds, Leeds LS2 9JT, U.K.

**Keywords:** cytokinins, end-of-flowering, flowering, reproductive architecture, shoot architecture, shoot branching

## Abstract

Cytokinin (CK) is a key plant hormone, but one whose effects are often misunderstood, partly due to reliance on older data from before the molecular genetic age of plant science. In this mini-review, we examine the role of CK in controlling the reproductive shoot architecture of flowering plants. We begin with a long overdue re-examination of the role of CK in shoot branching, and discuss the relatively paucity of genetic evidence that CK does play a major role in this process. We then examine the role of CK in determining the number of inflorescences, flowers, fruit and seed that plants initiate during reproductive development, and how these are arranged in space and time. The genetic evidence for a major role of CK in controlling these processes is much clearer, and CK has profound effects in boosting the size and number of most reproductive structures. Conversely, the attenuation of CK levels during the reproductive phase likely contributes to reduced organ size seen later in flowering, and the ultimate arrest of inflorescence meristems during end-of-flowering. We finish by discussing how this information can potentially be used to improve crop yields.

## Introduction

**Figure 1.**
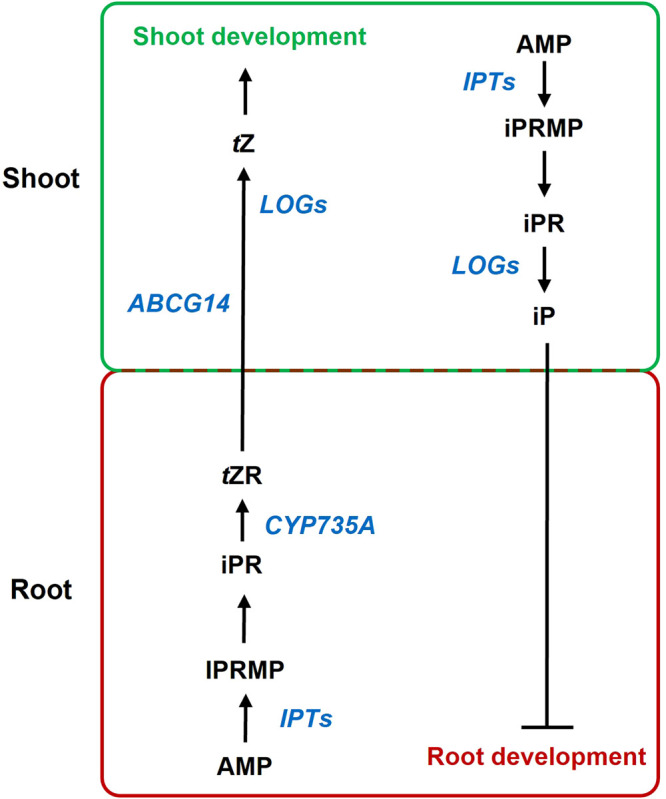
Cytokinin synthesis in angiosperms. Cytokinins are synthesised in both roots and shoots, and are transported both shoot to root (in the phloem), and from root-to-shoot (in xylem or xylem-associated cells). In the roots, ISOPENTENYL TRANSFERASE (IPT) and cytochrome P450 CYP7535A enzymes sequentially act on adenosine monophosphate (AMP) to form *trans*-Zeatin ribotide (*t*ZR), via isopentenyl adenine ribotide monophosphate (iPRMP) and isopentenyl adenine ribotide (iPR). None of these compounds have significant signalling activity. *t*ZR is transported to the shoot, facilitated by the transporter ABCG14, and is acted upon by LONELY GUY (LOG) enzymes to yield *trans*-Zeatin (*t*Z), which can activate the CK signalling synthesis pathway. In the shoot, isopentenyl adenine (iP), an active CK, is synthesised by from AMP, and transported to the root in the phloem. Thus, the predominant active CK in the root is iP synthesised in the shoot, while much of the active CK in the shoot is *t*Z synthesised in the roots. In this way, *t*Z and iP act as somewhat separate signals, moving in different directions and playing different roles to each other, despite sharing a synthesis and signalling pathway.

Plant hormones are extensively researched, and yet in many ways remain poorly understood; indeed, it often seems that the more we know, the more confusing these signals are. While we can try to ascribe meaning to them based on our experiments, understanding what these signals actually mean to plants themselves is much harder to discern. Cytokinin (CK), a hormone with an identity crisis, is a clear example of this trend, where the extensive progress in CK research over the last 20 years has only served to make it less clear what CK actually is. Far from being a monolithic signal, it seems very likely that the different structural CK types act as subtly different signals with different functions, moving in different directions within the plant, at least in angiosperms [1] (Figure 1). And while genetic analysis of CK certainly suggests it is important for normal plant growth and development, it no longer appears to be the equal and opposite of auxin, which — at the turn of millennium — was very much how it was conceptualised. However, classical ideas die hard, and much of the data now produced on CK is still interpreted in the context of old, pre-genomic frameworks, creating a disparity between what the data actually say, and how they are interpreted.

Shoot branching is a classic example of a process that was traditionally interpreted in the context of an auxin–cytokinin duality, and where the interpretation of data is still strongly influenced by this older idea [2,3], even when the data are not completely consistent with this idea [4]. The aim of this review is, therefore, to critically re-appraise the role of CK in the shoot architecture of plants, attempting to strip away pre-conceptions, and to focus on the most relevant evidence. It is not possible to cover every aspect of shoot architecture, and we will focus on the processes that determine the number, type and size of organ produced in the shoot, particularly during reproductive development.

## Cytokinins and shoot branching

The regulation of shoot branching is probably the best-known architectural role for CK, with a long history dating back to the pioneering work in the 1950s [5,6]. There is a wealth of ‘classical’ evidence that *exogenous* CK treatment promotes both the formation of axillary meristems (hereafter referred to as ‘buds’), and the subsequent outgrowth of buds to form branches [7]. This is supported by more recent studies to the same effect [8,9]. There is also abundant data that ectopically enhancing CK synthesis or signalling by transgenic means can also affect axillary bud formation and outgrowth [10,11,12]. However, while it is clear that endogenous CK does regulate the formation of axillary buds [13], there is puzzlingly little evidence that endogenous CK is really important for axillary bud outgrowth. In particular, a lack of clear branching phenotypes in loss-of-function CK mutants, which ought to be the ‘gold-standard’ evidence, should give us pause for thought.

**Figure 2.**
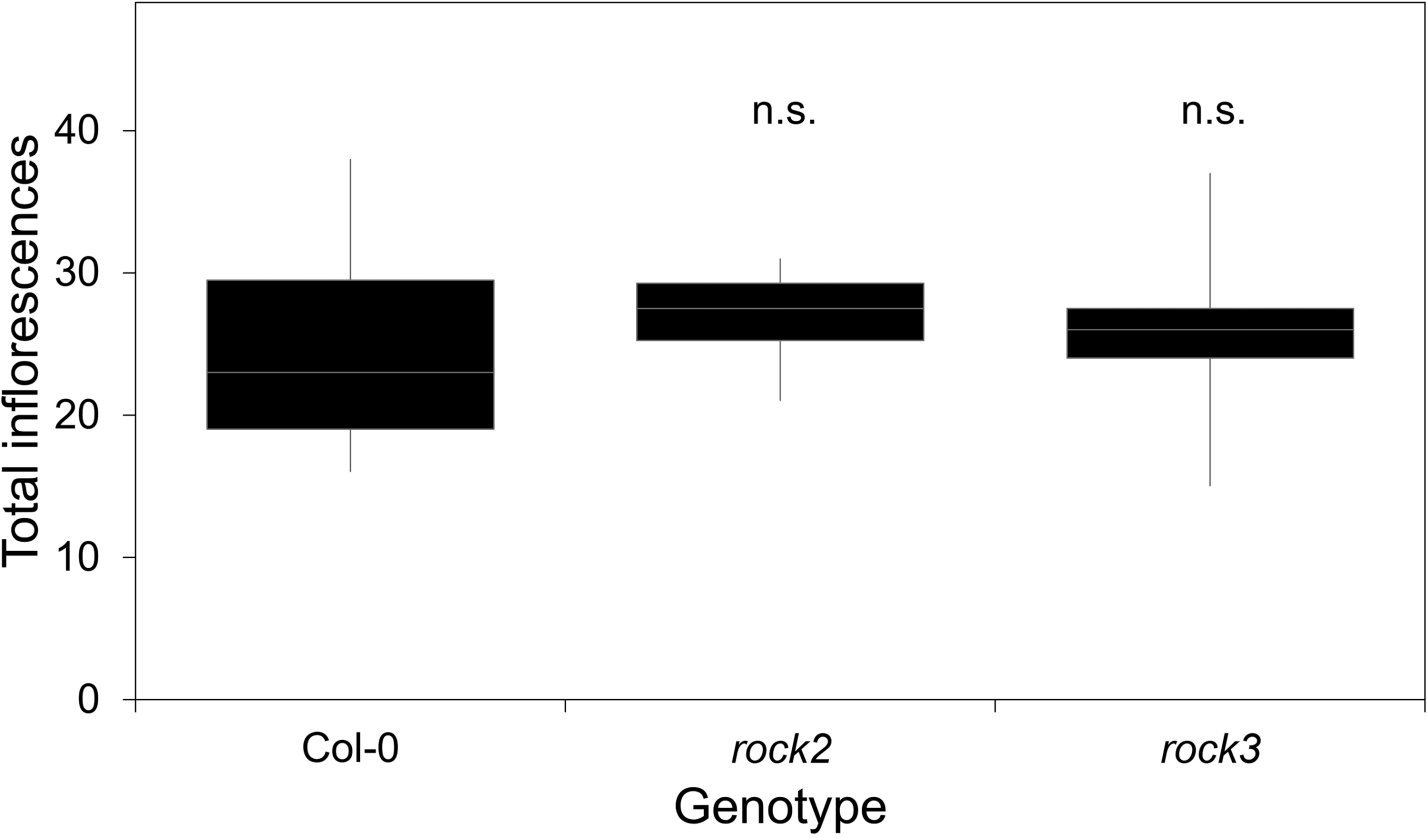
*rock* mutants do not exhibit a branching phenotype. Box plot showing total inflorescence number in *rock2* and *rock3* CK hypersensitive mutants relative to Col-0. Inflorescences were recorded following final arrest of the plant. Box shows the interquartile range, mid-line shows the median and whiskers show the maximum and minimum values. Neither *rock2* nor *rock3* is statistically different from the Col-0 wild-type (*P* = 0.682, ANOVA, Tukey honestly significant difference). *n* = 12.

The fact that CK synthesis/signalling components occur in large gene families, and the pleiotropy of higher order mutants thereof, has been blamed for this issue [4]. However, while this is certainly true of some families, it is not true for the CK receptor family, which only has three members in Arabidopsis (AHK2,3,4). Pleiotropy is certainly more of an issue, but mutants with severe deficiency in CK synthesis (e.g. *ipt3 ipt5 ipt7*) or signalling (e.g. *ahk2 ahk3*) in the shoot are far from uninterpretable. Rather, they are plants with small but well-formed shoots systems that produce fewer of every organ type (Figure 2). Neither issue is, therefore, truly a reason to dismiss the evidence from these mutants. However, finding published shoot branching data from CK mutants is difficult. For instance, three seminal studies performed in-depth characterisation of receptor single, double and triple mutants of Arabidopsis, but none reported on shoot branching [14,15,16]. More recently, Arabidopsis gain-of-function mutants in AHK2 (*rock2*) and AHK3 (*rock3*) with CK-hypersensitivity have been reported, but again, the shoot branching phenotype was not described [17]. Indeed, as far as we can tell, no published report has ever quantified the shoot branching phenotype of any of these mutants. In our hands, the *rock* mutants have no branching phenotypes (Figure 2), which is consistent with the effect of expressing the Arabidopsis *ROCK3* variant of *AHK3* in poplar [12]. Similarly, where branching phenotypes for other Arabidopsis CK mutants have been reported, they are invariably mild, consisting of small reduction in the number of branches, even in the fairly severe *ipt3 ipt5 ipt7* CK synthesis mutant [4,18]. Moreover, while the very severe *ahk2 ahk3 ahk4* and *ipt1 ipt3 ipt5 ipt7* mutants produce no branches, they are also generally much smaller plants, producing only a few flowers [15]. Since the purpose of branching in Arabidopsis is to produce more flowers, the reduction in branching in these lines is not necessarily because of a direct regulatory effect on bud outgrowth, but simply because the branches are not needed.

So, despite the strong evidence that exogenous CK influences bud outgrowth, there is relatively little evidence from Arabidopsis for a major role of endogenous CK in branching. However, a caveat should be mentioned here; Arabidopsis only undergoes visible branching during the reproductive phase, and technically all its branches are inflorescences whose behaviour and activity are governed by different regulatory dynamics than vegetative branches [19]. Inflorescence buds may be less CK sensitive than vegetative buds, and in species with vegetative branching, there might perhaps be clearer effects of CK on axillary bud outgrowth.

However, where currently available, this evidence is somewhat ambiguous. In tomato, lines overexpressing CYTOKININ OXIDASE2 (CKX2) (one of a class of enzymes that deactivates cytokinins), have reduced CK levels but strongly increased shoot branching, contrary to expectations [20]. Conversely, tomato *spl13* mutants have increased branching, which has been attributed to the up-regulation of CK synthesis in these lines [21], although the nature of the evidence here is not completely compelling. In rice, *cytokinin oxidase* (*ckx*) mutants are a major source of information. In early, reduced-expression lines, *ckx2* mutants were found to have increased tiller (vegetative branch) number [22,23], consistent with CK regulation of branching. However, more recently generated knockout *ckx2* lines have slightly decreased tiller number, especially in combination with *ckx1*, but have increased inflorescence (panicle) branching, resulting in more spikelets and grains per panicle [24]. Meanwhile, *ckx9* mutants consistently show increased tillering, especially in combination with *ckx4*, but have reduced panicle branching with reduced grain number per panicle [24,25]. RICE LATERAL BRANCHING (RLB) is a homeobox transcription factor that represses *CKX4*, and *rlb* mutants have strongly increased tillering, but this phenotype occurs against a background of strongly reduced CK levels, reduced panicle branching and grain number [26]. *CXK1*/*CKX2* and *CKX4* are all strongly expressed in the developing inflorescence, and is, therefore, unclear why their mutants should have such different phenotypes. A recently published mutant in the CK receptor HK4 offers similar ambiguity; it has decreased panicle branching, but increased tillering [27].

Interpreting these single time-point phenotypic data is made more difficult because there are homeostatic feedbacks between reproductive success and branching [19], such that mutants/treatments with reduced fertility often show compensatory increases in branching later in flowering [19,28], while increased fertility may also cause reduced branching. It is, therefore, unclear in these examples whether the changes in tillering are primary effects, or secondary effects of the changes in inflorescence branching (or vice versa). In this context, mutants in rice HK5 and HK6 CK receptors may offer some ‘*terra firma*’, and the strongest evidence that endogenous CKs promote shoot branching. *hk5 hk6* double mutants show both reduced tillering and reduced panicle branching, as would be expected if CK promotes branching [29]. However, this reassuring phenotype should not by itself explain away the general confusion in the rice data.

**Figure 3.**
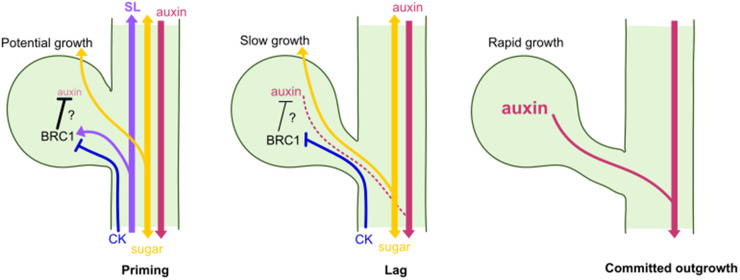
Three stages of bud outgrowth. Diagrammatic representation of three apparent stages of axillary bud outgrowth, highlighting the key regulatory events occurring in each. During priming, BRANCHED1 (BRC1) potentially acts directly to suppress auxin source strength in the bud, while CK (blue) inhibits BRC1 and moves buds towards activation. Strigolactones (SL) (purple) promote BRC1 expression. Sugars (yellow) are required for potential growth, and also repress BRC1 expression. During the lag phase, slow growth is initiated as auxin begins to canalise from the auxin source in the bud to the auxin sink in the main stem. CK may also promote this stage of bud growth. Committed outgrowth occurs when auxin canalisation is complete and rapid growth of the bud occurs, resulting a measurably outgrown branch. Arrows indicate positive relationships and direction of travel, while blunt arrows indicate inhibition. Question marks indicate proposed relationships.

In trying to explain the collective ambiguity in the evidence, it is important to remember that bud outgrowth is more complex than often presented. Typically, bud outgrowth is viewed as a binary switch between on/off, active/dormant states, and branching is measured as a binary present/absent trait. However, a range of work indicates that there are likely three distinct stages to bud outgrowth, each of which is independently regulated (Figure 3) [30,31,32,33]. In particular, it is worth noting that BRANCHED1, a master regulator of bud outgrowth, and known target of CK, regulates the early priming and lag phases of outgrowth, moving buds towards activation, but not the committed outgrowth of buds. It may, therefore, be the case that endogenous CK only plays an important role in the early phases of outgrowth, and therefore that CK mutants do not result in dramatic changes in the final elongated branch number. Higher levels of CK (i.e. from exogenous application) may be sufficient to drive buds into committed outgrowth, resulting in high branching, even though is not normally the effect of CK. Overall, more work is needed to understand the role of CK in shoot branching, rather than simply accepting the received wisdom that it is a key regulator.

## Cytokinins and inflorescence meristem activity

There is rather better genetic evidence for a key role of CKs in the development of inflorescences, and particularly the size and rate of activity of the inflorescence meristem (IM). This is perhaps unsurprising, since it is well-established that CKs positively regulate the activity of vegetative shoot meristems in Arabidopsis (reviewed in [34]). In Arabidopsis, *ahk2 ahk3* double mutants have smaller IMs that produce flowers at a slower rate [15,35], while *ipt3 ipt5 ipt7* triple mutants have a similar phenotype [36]; the IMs in the more severe *ahk2 ahk3 ahk4 and ipt1 ipt3 ipt5 ipt7* mutants collapse very soon after flowering begins [15,36]. Conversely, *ckx3 ckk5* double mutants have larger IMs that produce flowers at faster rate [37]. The same effects are seen in *ckx3 ckx5* mutants of *Brassica napus*, confirming the generality of the effect [38]. IM size in the rice *hk4* mutant is also reduced, consistent with this effect [27]. The effect of soil nitrate on IM size/activity is mediated through CKs, such that under low nitrate, less CK is synthesised in the roots and transported to the shoots, resulting in a smaller IM with slower activity [39]. Thus, CKs act as a mechanism to couple reproductive effort to soil nutrient availability.

## Cytokinins and floral meristem and organ size

Although nowhere directly quantified, it seems that the effects of CK on the IM are also seen in floral meristems. The Arabidopsis *ckx3 ckx5*, *rock2* and *rock3* mutants all have greatly enlarged flower size, as do the *B. napus ckx3 ckx5* mutants, presumably due to enlarged floral meristems earlier in development. In *rock2* and *rock3*, there is no change in IM size [35], so this change in flower size is not simply a consequence of a larger IM. The *ahk2 ahk3 ahk4* triple mutants appear to have slightly reduced flower size, but flower structure is largely normal. However, in rice *hk5 hk6* mutants, the flower structure is severely affected, with a reduction in floral organ number (mostly obviously stamen number) and size [29]. This is consistent with the phenotype observed in the rice *lonely guy* mutant, which lacks a key enzyme in CK synthesis [40]. Thus, the available data do support a clear role for CK in the development of flowers.

## Cytokinins and fruit/seed development

The effects of CK on flower development carry over into the subsequent development of fruit and seed. The *ckx3 ckx5* mutants of both Arabidopsis and *B. napus* produce larger fruit, with substantially more ovules per gynoecium [37,38]. These gains in ovule number do not completely translate into increased seed set, particularly in *B. napus*, a consequence of reduced self-fertility caused by increased elongation of the gynoecia relative to the stamens. However, due to the increase in flower numbers, the mutants produce seed yields ∼55% (Arabidopsis) and ∼30% (*B. napus*) higher than the corresponding wild-type [37,38]. In most crop species, the formation of yield can be impacted in many different ways, such as increasing individual seed weight, increasing the number of branches, tillers or panicles or increasing the number of seeds per flower. While in principle these are effective methods for altering yield, gains in one parameter typically result in a compensatory trade-offs in other parameters [41]. Interestingly, these *ckx* mutants increased seed yield with no apparent trade-off in seed weight, and show that manipulating CK homeostasis during reproductive development may be a promising approach to improve crop yields.

Unlike *ckx* mutants, *rock2* mutants typically produce smaller fruits than wild-type despite producing larger flowers, apparently due to an ever bigger mismatch between the length of the gynoecia and stamens [17]. However, the individual seeds are significantly larger in *rock2* mutants. While the exact mechanism for this is unclear, *rock2* shows enhanced cellular proliferation and delayed senescence [17], either of which could potentially increase seed mass, through a greater number of cells or through prolonged availability of nutrients during ripening. Seed mass is similarly increased in the *ahk2 ahk3 cre1/ahk4* triple mutant due to increased embryo size, controlled by the maternal and endosperm genotypes [16]. Overall, these data show a crucial role of CKs in controlling seed size.

Arabidopsis shows a decline in fruit length over time, with shorter fruits supporting fewer seeds at the ends of the inflorescences [42]. Given the above data, and considering CK signalling in the inflorescence declines during the reproductive phase (below) [35], it is likely that this decline in fruit size and seed number is brought about at least in part by declining CK levels. This effect is more severe in *B. napus*, with many later-setting fruits simply aborting [43]. In the desert plant *Aethionema arabicum* (also in the Brassicaceae), a more binary version of the same phenomenon occurs. *A. arabicum* produces dimorphic fruits; a larger, dehiscent fruit, and a smaller, non-dehiscent fruit containing a single seed. The large morph tends to develop earlier, with the majority of later-developing fruits being the small morph [44]. However, treatment with CK is sufficient to increase the proportion of large morph fruit, suggesting that CK levels during flowering may control fruit morph [45]. A similar trend is seen in wheat ears, with lager seeds developing in the middle of the ear, decreasing in size towards the top and bottom [46], following the temporal order of grain development [47]. While more work would be needed to test these ideas, the evidence tentatively suggests that altering CK dynamics during the reproductive phase represents a promising avenue to increase crop productivity by increasing seed size/number in later-developing flowers.

## Cytokinins and end-of-flowering

Recent work also suggests a role for CK in determining the timing of the end-of-flowering in Arabidopsis. End-of-flowering has been a rather neglected area, but recent work has defined the developmental basis for this in Arabidopsis [35]. This consists of the arrest of IMs, mid-way through the visible period of flowering, followed by ‘floral arrest’, a block on further flower development that results in the production of a cluster of ∼15 unopened floral buds on each inflorescence [35]. Both of these phenomena seem to be associated with CK signalling.

The link between CK and IM activity has already been discussed. WUSCHEL (WUS), a homeobox domain transcription factor, is required for the maintenance of IM activity [48], and is regulated through CK signalling [49]. Conversely, regulated IM arrest is associated with the reduction in the expression of the *TCSn:GFP* reporter in the IM [35,50], with a concomitant reduction in *WUS* expression [51,52]. Mirroring the reduction in *TCSn:GFP* expression, expression of type-A *ARABIDOPSIS RESPONSE REGULATOR5* (*ARR5*) and *ARR7*, which are up-regulated in response to CK, also declines in the run-up to IM arrest [35]. Consistent with this, *rock2* mutants show prolonged flowering, due to prolonged IM activity [17,35]. As a result, *rock2* produces a greater number of floral primordia than wild-type, although at the same rate, and produces more fruit. However, some caution in interpreting these data is required, since *ahk2 ahk3* IMs remain active for the same length of time as wild-type, while producing flowers at a slower rate.

Cytokinin also seems to regulate floral arrest, independently of IM arrest. Unlike wild-type, *rock2* mutants open almost all flowers they produce, leaving no bud cluster [35]. The same effect is seen in *rock3* mutants, which do not show delayed IM arrest, emphasising that these are separable effects [35]. Since the developmental block in floral arrest occurs at floral stage 9, long after the floral meristem arrests, this effect of CK may be independent of its effect on floral meristem activity, although more work is required to really understand floral arrest.

These data imply that the end-of-flowering in Arabidopsis requires the strong reduction in CK levels in inflorescences in order for IM and floral arrest to occur. This reduction might occur as a natural consequence of the progressive expansion of the reproductive system. During flowering, plants initiate more and more inflorescences, each of which generates more and more flowers, fruits and seeds, creating a rapidly expanding number of organs [19,35,53]. If these organs are sinks for root-derived *trans*-Zeatin (*t*Z) type CK, and if *t*Z supply is relatively constant, then dilution of *t*Z across the shoot system will occur during flowering, such that eventually a critical threshold is reached, triggering IM and floral arrest. Consistent with this, the removal of inflorescences and fruits is sufficient to maintain *TCSn:GFP* expression in IMs, and prolong flowering, but not in the *ahk2 ahk3* mutant [35]. This would (if validated) be a simple and robust system allow the duration of flowering to be co-ordinated with both soil nutrient levels and current reproductive success (measured via sink number).

## Conclusion

**Figure 4.**
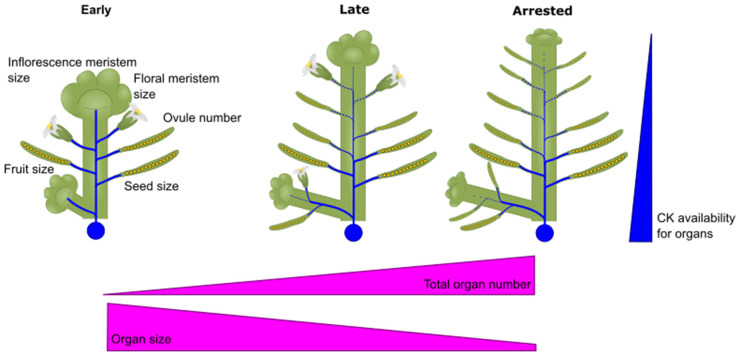
Effects of cytokinin on development throughout flowering. Diagrammatic representation of CK effects on floral development over time. Early in the plant development, the CK (blue) source is sufficient to maintain the growth of all organs, including inflorescence meristems (IMs), flowers and developing fruits and seeds. Later in flowering flowering, CK availability declines in the IMs, reducing their size. Floral meristem size, fruit size, ovule number and seed size all begin to decline in later fruits. When CK levels drop below a critical threshold, the IMs arrest, and no new flowers are initiated.

The data reviewed here suggest that CK has a profound impact on reproductive development in angiosperms, increasing the size of both inflorescence and floral meristems, with knock-on effects on ovule number, fruit and seed size (Figure 4). However, the natural attenuation of CK levels and/or signalling during reproductive development means that later-initiated flowers are less productive than they might be, either forming smaller fruits, not completing development, or completely aborting (Figure 4). Thus, boosting CK signalling during reproductive development represents a very appealing option to explore for increasing crop yield, with demonstrable results already shown in *B. napus* [38]. In mutants with increased CK signalling during reproductive development, only the mismatch between staemen and gynoecium elongation prevents even higher yields being realised.

Reproductive development is not one of the classically defined roles of CK, but the genetic data are unambiguous in their support of it. While genetic data are not without limitations, and can be misinterpreted, the collective strength of the data here is convincing. Conversely, shoot branching, which is a classically defined role of CK from the ‘spray-and-pray’ era of plant hormones, is relatively poorly supported by genetic data. While it seems unlikely that CK does not regulate branching in some manner, careful consideration of the data does suggest more work is required to establish exactly how CK does this. While classical data should never be dismissed arbitrarily, nor should they be over-esteemed, and the data presented here suggest that a new framework for CK and shoot architecture is long overdue.

## Perspectives

- CKs are important plant hormones with strongly promotive effects on growth, but whose effects are often misunderstood.
- CK has clear roles in producing ‘bigger and better’ reproductive shoot system of plants when conditions are good. However, the classically defined role for CK in shoot branching needs re-evaluating.
- Manipulation of cytokinin synthesis or signalling in crop plants could boost yield by increasing fruit and seed numbers.

## Open Access

Open access for this article was enabled by the participation of the University of Leeds in an all-inclusive *Read & Publish* agreement with Portland Press and the Biochemical Society under a transformative agreement with JISC.

## Abbreviations

CK: cytokinin
IM: inflorescence meristem
